# Comparison of a mycobacterial phage assay to detect viable *Mycobacterium avium* subspecies *paratuberculosis* with standard diagnostic modalities in cattle with naturally infected Johne disease

**DOI:** 10.1186/s13099-021-00425-5

**Published:** 2021-05-06

**Authors:** Robert J. Greenstein, Liya Su, Irene R. Grant, Antonio C. G. Foddai, Amy Turner, Jason S. Nagati, Sheldon T. Brown, Judith R. Stabel

**Affiliations:** 1 Department of Surgery, James J. Peters Veterans Affairs Medical Center, Bronx, NY USA; 2 Laboratory of Molecular Surgical Research, James J. Peters Veterans Affairs Medical Center, Bronx, NY USA; 3 Institute for Global Food Security, School of Biological Sciences, Queen’s University Belfast, Belfast, UK; 4 Johne’s Disease Research Project USDA-ARS-NADC, Ames, IA USA; 5 Department of Medicine, Columbia University Medical Center, New York, NY USA; 6 Infectious Disease Section, James J. Peters Veterans Affairs Medical Center, Bronx, NY USA; 7 Department of Medicine, Icahn School of Medicine at Mt. Sinai, New York, NY USA

**Keywords:** Mycobacterium avium subspecies paratuberculosis (MAP), Phage assay, Quantitative PCR, Johne disease, Crohn disease, Peripheral blood mononuclear cells (PBMCs), Mycobacteriophage

## Abstract

**Background:**

*Mycobacterium avium* subspecies *paratuberculosis* (MAP), the cause of Johne disease, is a slow growing mycobacterium. Viable MAP detection is difficult, inconstant and time-consuming. The purpose of this study was to compare a rapid phage/qPCR assay performed on peripheral blood mononuclear cells (PBMCs) with three standard methods of MAP detection: fecal MAP PCR; plasma antigen-specific IFN-γ & serum MAP ELISA hypothesizing that, if sensitive and specific, Johne animals would be positive and Control animals negative. We studied a well characterized herd of Holstein cattle that were naturally infected with MAP and their Controls.

**Results:**

With phage/qPCR 72% (23/32) of Johne and 35% (6/17) of Controls were MAP positive. With fecal PCR 75% (24/32) of Johne and 0% (0/17) of Controls were MAP positive. With plasma antigen-specific IFN-γ 69% (22/32) of Johne and 12% (2/17) of Controls were MAP positive. With serum MAP ELISA, 31% (10/32) of Johne and 0% (0/17) of Controls were MAP positive. When phage / qPCR and fecal PCR results were combined, 100% (32/32) Johne and 35% (6/17) of Control animals were MAP positive. Younger Control animals (1–3 years) had significantly fewer plaques (25 ± 17 SEM) than older Controls (4–12 years) (309 ± 134 p = 0.04). The same trend was not observed in the Johne animals (p = 0.19).

**Conclusions:**

In contrast to our hypothesis, using the phage/qPCR assay we find that viable circulating MAP can rapidly be detected from the blood of animals infected with, as well as those in the Control group evidently colonized by MAP. These data indicate that the presence of viable MAP in blood does not necessarily signify that an animal must of necessity be demonstrably ill or be MAP positive by standard diagnostic methods.

## Background

Johne disease [1], a chronic inflammatory intestinal disease, is caused by *Mycobacterium avium* subspecies *paratuberculosis* (MAP). Johne disease causes considerable financial loss to the agricultural community [2, 3] and is present worldwide [4]. There is increasing concern that MAP may be zoonotic, contributing to inflammatory bowel disease in humans, particularly Crohn disease [5–12]. Although controversial, other human diseases have been considered to be associated with MAP, either directly or consequent to molecular mimicry [13–16].

Many mycobacteria including MAP, *Mycobacterium leprae* and *Mycobacterium tuberculosis* grow slowly. The “gold standard” for the detection of MAP is culture, but this is both time consuming and difficult [17]. In cattle, primary isolation requires a minimum of 8 weeks, and in humans with Crohn disease up to 18 months [18]. When detectable by culture, this results in tardiness in making reliable diagnosis. Although not indicative of viability, when present in abundance, MAP qPCR is the most effective method of detecting the presence of MAP DNA [19]. Accordingly, it is desirable to develop reliable and rapid methods to identify the presence of viable MAP.

Mycobacterial phage assays, that provide rapid results, require the presence of viable mycobacteria that contain a cell wall that permits the phage to attach and then enter the mycobacterium. The phage then subverts the host cell’s metabolic process, producing copies of itself. This ultimately results in lysis of the infected cell with release of progeny phages in addition to host DNA. A mycobacterial phage/qPCR assay that identifies and quantifies MAP in bovine blood, specifically in peripheral blood mononuclear cells (PBMCs), would be a powerful addition to expedite the diagnosis of viable MAP in infected eukaryotes. Such assays have been used to diagnose the presence of viable MAP in bovine milk and feces [20, 21] in naturally infected cattle with Johne disease [22] and a herd that had been experimentally inoculated with MAP [23] and most recently in humans with Crohn disease and some of their controls [24].

The purpose of this study was to compare results of standard MAP diagnostic modalities (MAP fecal PCR, serum MAP ELISA and plasma antigen-specific MAP IFN-γ,) with a mycobacterial phage amplification assay followed by qPCR [20, 23, 25–27] that permits rapid (< 24 h) diagnosis of viable MAP. Animals are from a herd of naturally MAP infected Holstein dairy cattle and their (nominally) non-infected controls, housed by the USDA at their facility in Ames, Iowa USA. Early in this index, initially blinded, plaque/qPCR study there were intriguing and unexpected results. Positive MAP signal was observed in some Control animals and negative MAP in some Johne animals. Consequently, we converted to an open label study comparing Johne animals with Controls.

## Results

### Phage assay: Determining satisfactory mycobacteriophage virucidal activity

We evaluated D29 from two sources. Failure to obtain satisfactory results with the negative control with our initially sourced D29, led us to compare different vortex times, two different batches of FAS, and a second source of D29. The FAS was determined not to be the problem (Table 1). We could not completely kill unincorporated D29 obtained from the Pittsburgh source. The alternative QUB D29 strain gave satisfactory negative controls and was used throughout subsequent experiments.

**Table 1:** Comparison of two strains of D29 and two sources of ferrous ammonium sulfate (FAS)

		Plaque #	
	D29 pfu/ml	D29: U. Pittsburgh	D29: Queens U. Belfast
Positive Control	10^2^	92	98
	10	10	17
	1	1	1
FAS USA	10^8^	14	0
	10^8^	21	0
	10^8^	23	0
FAS EUROPE	10^8^	20	0
	10^8^	23	0
	10^8^	23	0

**Fig. 1:**
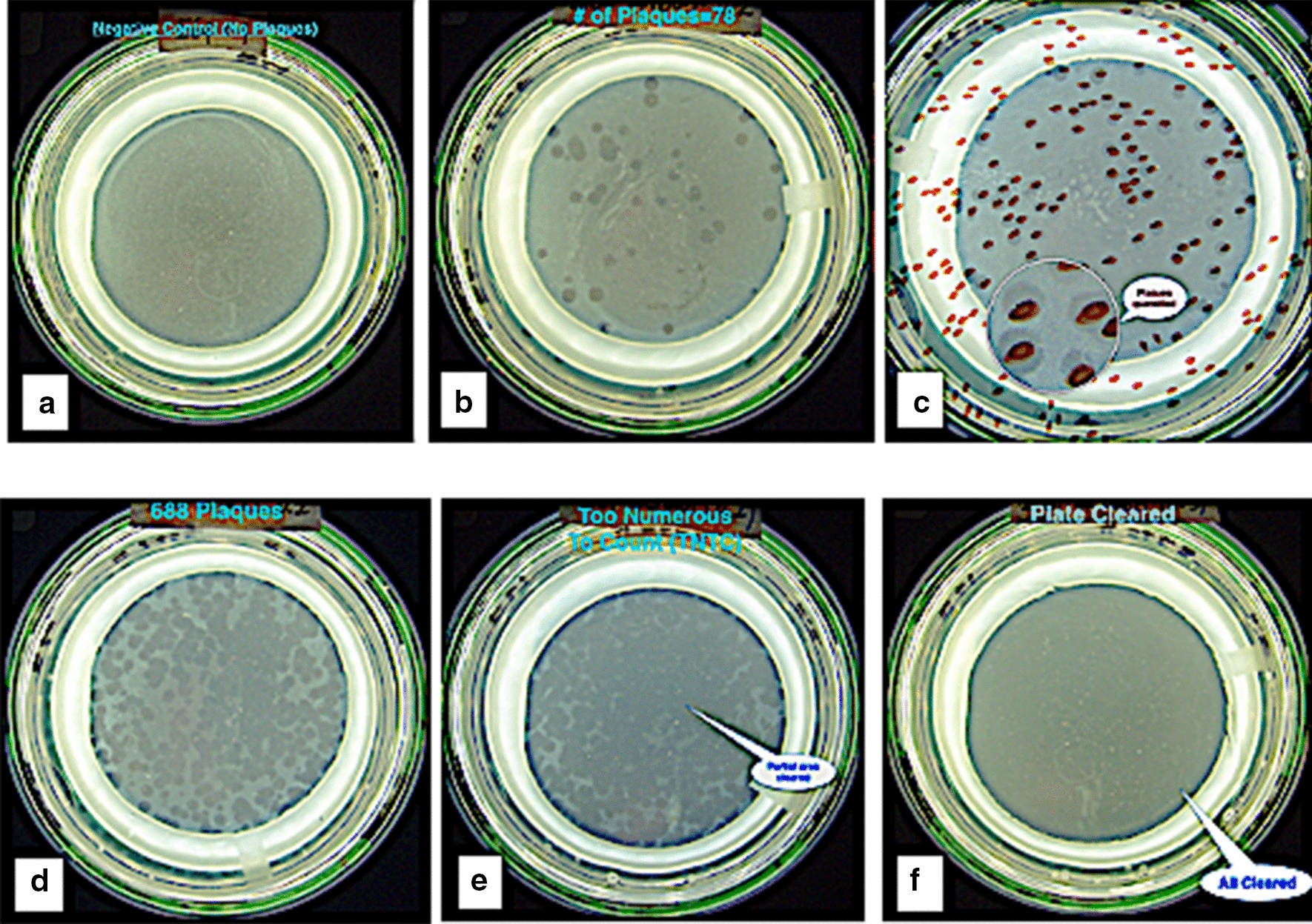
Photographs of representative plaques. **a** A negative control. **b** Show the appearance of a small number of plaques; in this case taken from 10% of the buffy coat of 8 ml of blood. **c** Shows how the plaque numbers are obtained. Using the Stuart Colony Counter, each time a mark is made on the bottom of the petri dish, as the dish is depressed, the count is automatically tabulated. **d** A plate where the plaque count is 688 plaques, determined using the Stuart colony counter as demonstrated in panel C. **e** A plate where the number of plaques are “Too Numerous To Count.” In this particular case the inoculum was from 10% of the buffy coat. **f** 25% of the buffy coat from the same blood sample as shown in “**e**” was inoculated. In this plate the plaques are so numerous that the entire plate has been “Cleared”. No discrete plaques at all are discernable. Compare this appearance with that of the hazy appearance of the Control plate (Panel “A”) which is the appearance of the *M. smegmatis* lawn. (see Materials and methods)

A comparison of two sources of mycobacteriophage D29, (see Materials and methods) with two sources of the virucide, ferrous ammonium sulfate (see Materials and methods). Satisfactory viricidal action was only achievable with D29 sourced from Queens University Belfast, (see example in Fig. 1a). As a consequence, the QUB D29 was used in all subsequent experiments. PFU: Plaque Forming Units

Preliminary qPCR Results: Identification of quencher of choice. We next had to perform IS900 qPCR on DNA isolated from individual plaques (see Fig. 1b, c).

**Fig. 2:**
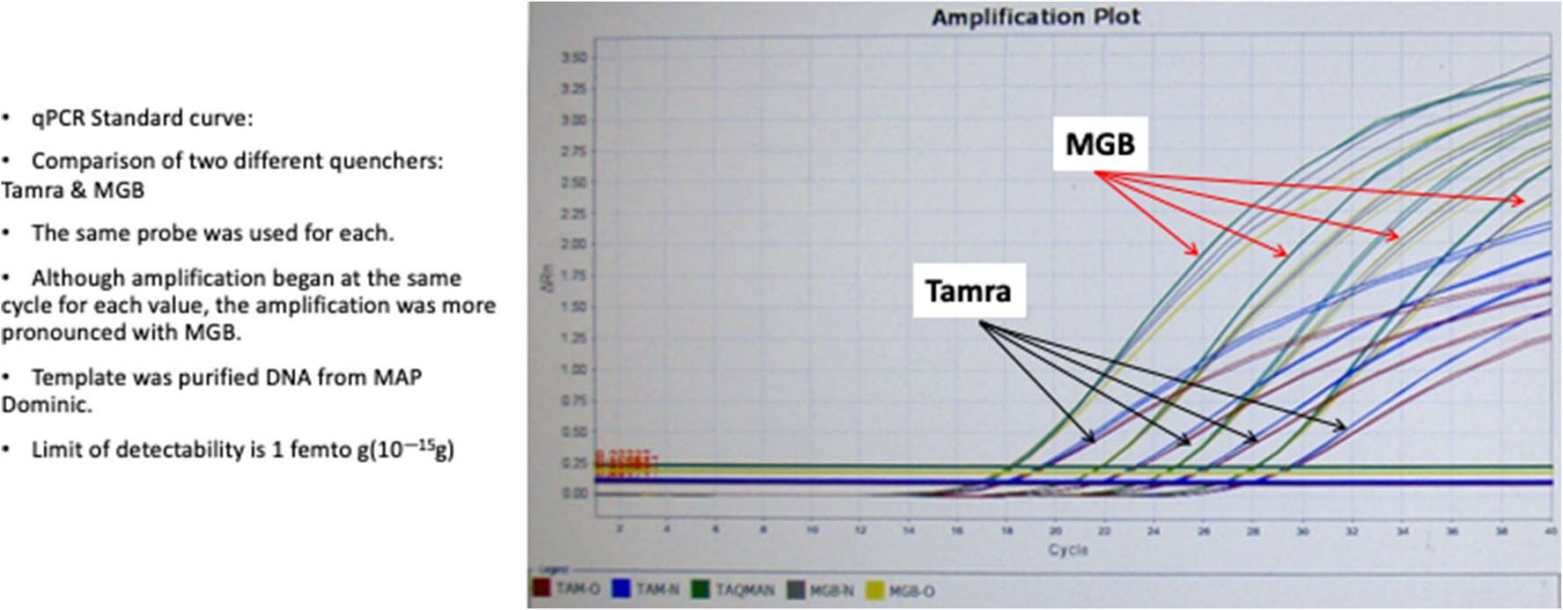
A screen shot of a qPCR comparing two quenchers: TAMRA and MGB. Studied are serial dilution of MAP DNA isolated from pure culture of “Dominic” (ATCC 43545). Note the more pronounced amplification with MGB

We first compared two different quenchers: the recommended TAMRA [27] with the manufacturer recommended quencher MGB. Both quenchers had the same initial level of detectability, however, the fluorescent release was more pronounced with MGB (see Fig. 2). Accordingly, we subsequently used MGB as the quencher.

### Limit of detectability

The limit of detectability of DNA purified from MAP Dominic ATCC 43545 and quantified by Qubit® was 1 fg (1 × 10^−18^ g).

Summarized in Table 2 are the results of all the blood (ELISA, IFN-γ & Phage/qPCR) and fecal PCR tests. In the Control group there were no positives with ELISA or fecal PCR. In contrast, positives are found in 12% of Control animals by IFN-γ and in 35% by phage/qPCR. In the Johne animals, 31% were ELISA positive, 69% IFN-γ positive, 72% phage/qPCR positive and 75% fecal PCR positive.

**Table 2:** MAP: Blood ELISA, INFγ, plaque qPCR & fecal PCR

MAP	Control				Johne			
	Pos		Neg		Pos		Neg	
	%	#	%	#	%	#	%	#
ELISA	0	0/17	100	17/17	31	10/32	69	22/32
IFNγ	12	2/17	88	15/17	69	22/32	31	10/32
Fecal PCR	0	0/17	100	17/17	75	24/32	25	8/32
Phage / qPCR	35	6/17	65	11/17	72	23/32	28	9/32

A summary of standard test results compared with the data from the phage/qPCR experiments. In the Control animals the ELISA and fecal PCR have no positives, whereas IFN-γ was 12% and phage / qPCR 35% positive. ELISA (31%+) is least sensitive in the Johne animals. IFN-γ (69%+), fecal PCR (75%+) and phage / qPCR (72%+) the most sensitive

Table 3 presents the individual test results for all (n = 17) Control animals.

**Table 3:** Control cow data

Animal #	ELISA+/−	IFNγ+/−	Fecal PCR+/−	Plaque qPCR +/−
1c	−	−	−	+
2c	−	−	−	+
3c	−	−	−	−
4c	−	−	−	+
5c	−	−	−	+
6c	−	−	−	+
7c	−	−	−	−
8c	−	−	−	+
9c	−	−	−	−
10c	−	−	−	−
11c	−	+	−	−
12c	−	−	−	−
13c	−	−	−	−
14c	−	−	−	−
15c	−	−	−	−
16c	−	+	−	−
17c	−	−	−	−
% positive (# positive/total tested)	0% (0/17)	12% (2/17)	0% (0/17)	35% (6/17)

Animals #1c-12c are from the Johne control herd. Animals #13c-17c are from a separate herd, the Control for a mastitis study. Note all six phage/qPCR positive animals are in the Johne control herd. Two animals were IFN-γ positive as were five with phage/qPCR. None of IFN-γ and phage/qPCR positives overlapped

Table 4 presents the individual test results for all (n = 32) Johne animals.

**Table 4:** Johne data

Animal #	ELISA+/−	IFNγ+/−	Fecal PCR+/−	Phage/qPCR +/−
1j	−	+	+	+
2j	−	+	−	+
3j	−	+	+	−
4j	−	−	−	+
5j	−	−	+	−
6j	−	−	−	+
7j	−	+	−	+
8j	−	+	−	+
9j	−	+	+	+
10j	+	+	+	+
11j	−	+	+	−
12j	−	+	+	−
13j	−	+	−	+
14j	−	+	+	+
15j	−	−	+	+
16j	−	+	+	−
17j	−	+	+	+
18j	−	+	+	+
19j	+	−	+	−
20j	−	+	+	−
21j	+	+	+	+
22j	+	−	+	−
23j	−	+	+	+
24j	−	+	+	+
25j	−	+	−	+
26j	+	−	+	+
27j	−	−	+	+
28j	+	−	+	−
29j	+	−	−	+
30j	+	+	+	+
31j	+	+	+	+
32j	+	+	+	+
% positive (# positive/total tested)	31% (10/32)	69% (22/32)	75% (24/32)	72% (23/32)

ELISA is the least sensitive at demonstrating MAP infection. IFN-γ, Fecal PCR and phage/qPCR have comparable sensitivity. Of the ten animals that were positive on ELISA, 5 were positive by all four methods. Two of the remaining ELISA positives were positive on Fecal PCR

### Determining plaque count

The mean plaque number isolated from the PBMC’s of 0.8 ml blood (i.e.10% of original 8 ml blood) for Control animals was 168±71 PFU and for Johne 194 ± 95 PFU (SEM: p = NS.)

**Fig. 3:**
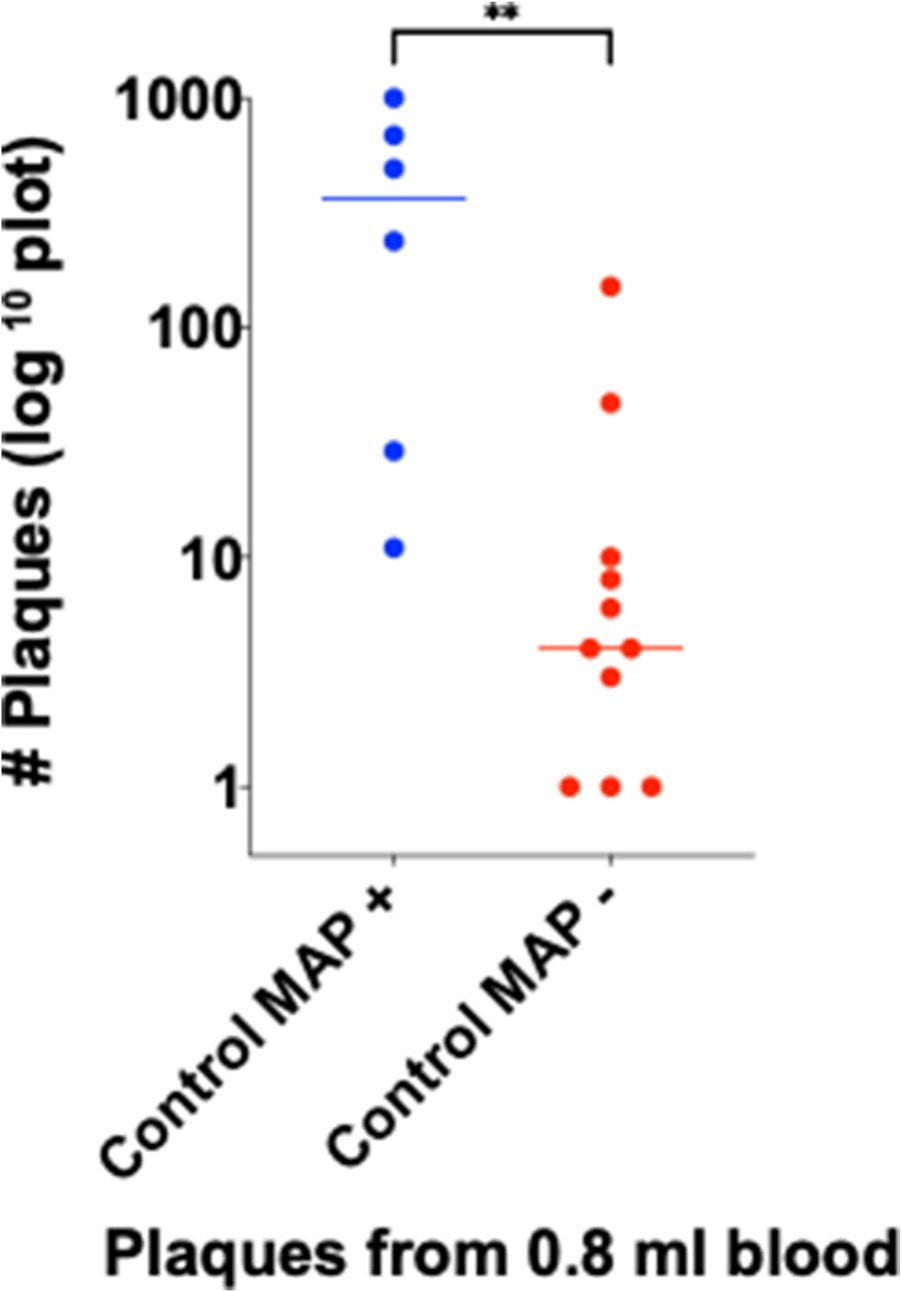
A comparison of Control animals that are MAP positive or negative by the phage/qPCR assay. There are significantly fewer plaques in the plates of the Phage/qPCR assay in the MAP negative controls (Mann–Whitney two tailed p = 0.0018; displayed as **)

Table 5 shows cumulative data for plaque # and qPCR results (Fig. 3).

**Table 5:** Age, plaque numbers and qPCR (+/− IS900) for each animal

	Control					Johne			
#	Age (years)	Plaque #	qPCR Pos/Neg	Total assay # (# Pos)	#	Age (years)	Plaque #	qPCR Pos/Neg	Total assay# (# Pos)
1c	8	11cal	+	4 (1)	1j	8	27	+	7 (6)
2c	8	1000est	+	9 (4)	2j	12	312	+	5 (1)
3c	12	3	−	4 (0)	3j	12	139	−	8 (0)
4c	12	238	+	4 (1)	4j	9	1000est	+	4 (2)
5c	5	688	+	8 (7)	5j	9	25	−	8 (0)
6c	5	29	+	11 (3)	6j	9	30	+	12 (2)
7c	4	10	−	4 (0)	7j	5	189	+	4 (2)
8c	4	492	+	16 (3)	8j	5	98	+	12 (3)
9c	3	1	−	4 (0)	9j	13	14	+	4 (1)
10c	3	151	−	5 (0)	10j	4	5	+	4 (1)
11c	3	47	−	4 (0)	11j	4	8	−	8 (0)
12c	1.3	4	−	4 (0)	12j	4	545	−	5 (0)
13c	2	1cal	No DNA	No DNA	13j	4	129	+	4 (2)
14c	2	8cal	−	4 (0)	14j	3	78	+	12 (1)
15c	2	1cal	No DNA	No DNA	15j	3	38cal	+	1 (1)
16c	2	4	−	4 (0)	16j	3	59	−	12 (0)
17c	1.3	6	−	4 (0)	17j	3	26	+	4 (1)
					18j	3	214	+	5 (1)
					19j	>11	48	−	12 (0)
					20j	7	449	−	3 (0)
					21j	5	91	+	14 (7)
					22j	5	135	−	2 (0)
					23j	5	308	+	15 (1)
					24j	5	321	+	14 (3)
					25j	4	97	+	11 (3)
					26j	11	145	+	4 (4)
					27j	10	1000est	+	8 (5)
					28j	5	359	−	2 (0)
					29j	9	229	+	12 (3)
					30j	7	17	+	4 (2)
					31j	5	64	+	12 (3)
					32j	3	5	+	4 (4)
		Mean = 159±71	+= 35% (6/17)				Mean = 194±44	+= 72% (23/32)	

The count number of plaques (mean ± SEM) presented for 10% of the PBMC’s harvested from 8 ml whole blood following Ficoll gradient. When no plaques were seen, the number presented as “cal” (see Materials and methods). The number of individual qPCR replicates are presented as the total for a given animal with the number of positives in adjacent parentheses (see Materials and methods). “No DNA” indicates that there were so few plaques, that no DNA was extracted. In the Control animals, MAP + animals had significantly more plaques than MAP- animals (Fig 3)

**Fig. 4:**
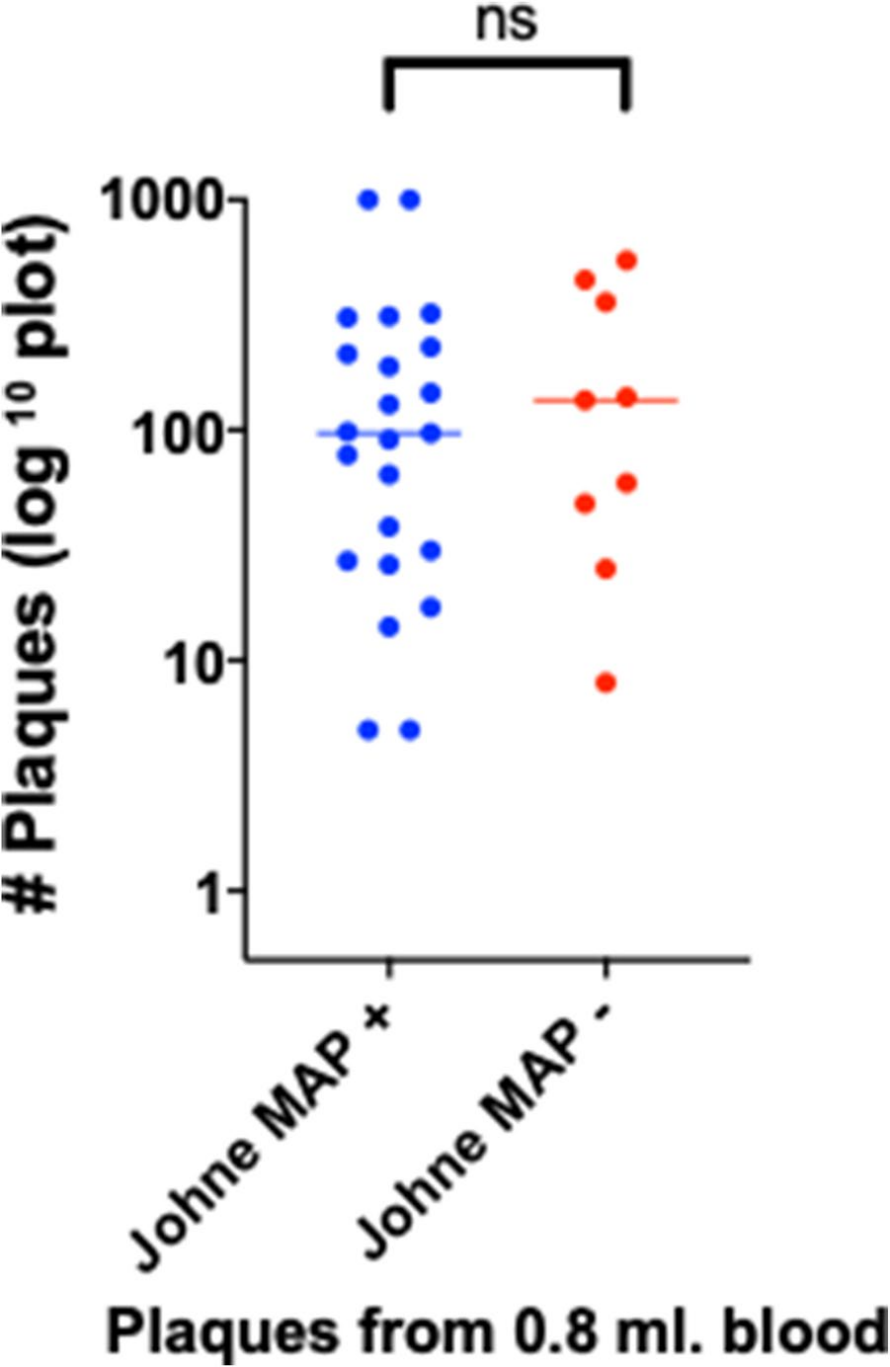
A comparison of number of plaques between Johne animals that are MAP positive or negative by the phage/qPCR assay. There is no difference between the number of plaques in the two groups (Mann–Whitney two-tailed p = 0.7038)

In contrast, amongst the Johne animals there was no difference between the MAP+ and – animals (Fig 4).

**Fig. 5:**
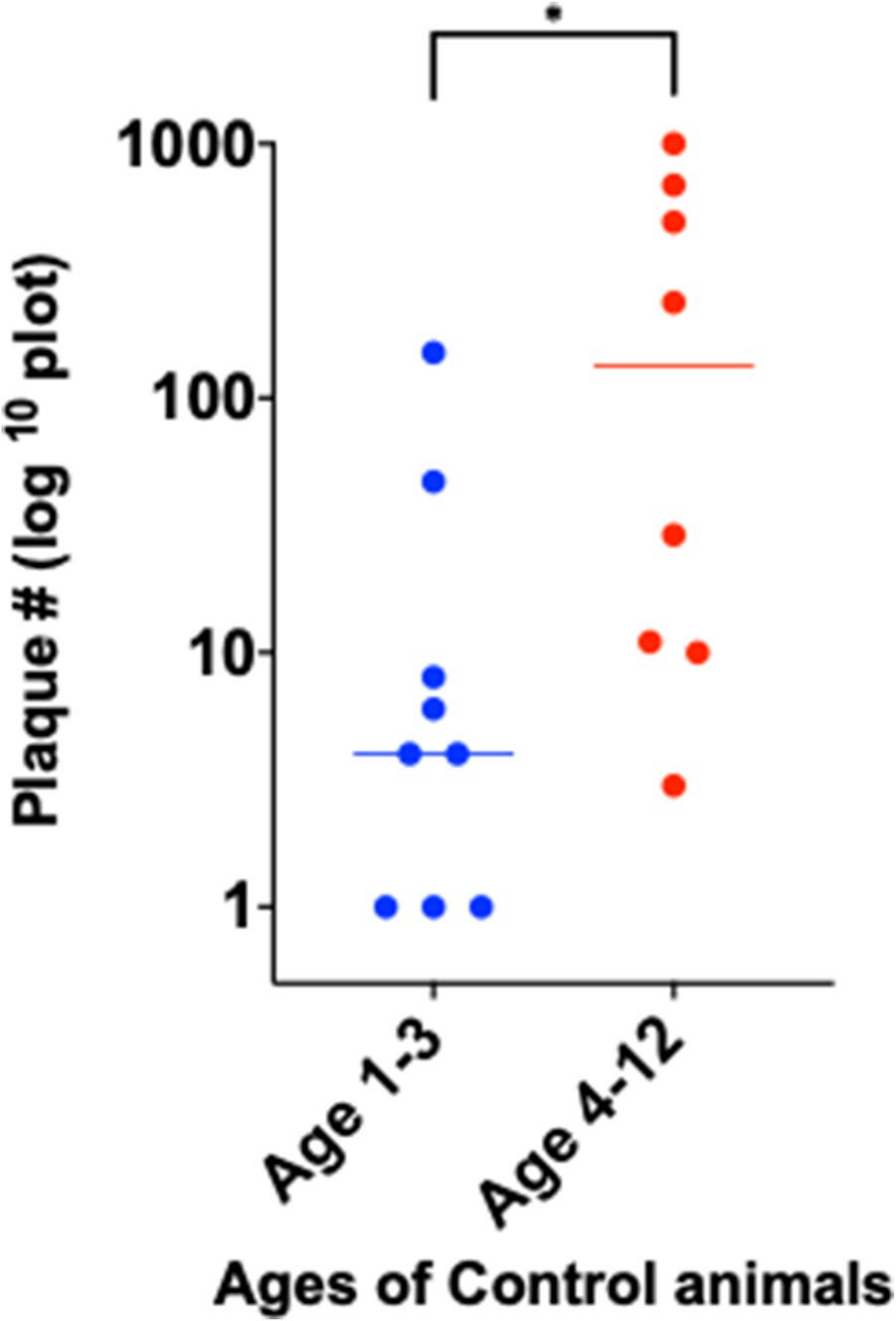
When Control animals are stratified by ages 1–3 (53%; 9/17). and 4–12 (47%; 8/17) years, the younger Control animals have significantly fewer plaques (25 ± 16) than the older animals (308 ± 134: Mean ± SEM: p = 0.04; unpaired two tailed t-test.)

When the Control animals were stratified by age groups (1–3 and 4–12 years), there were significantly fewer plaques (indicating total mycobacterial burden) in the younger animals (Fig 5).

**Fig. 6:**
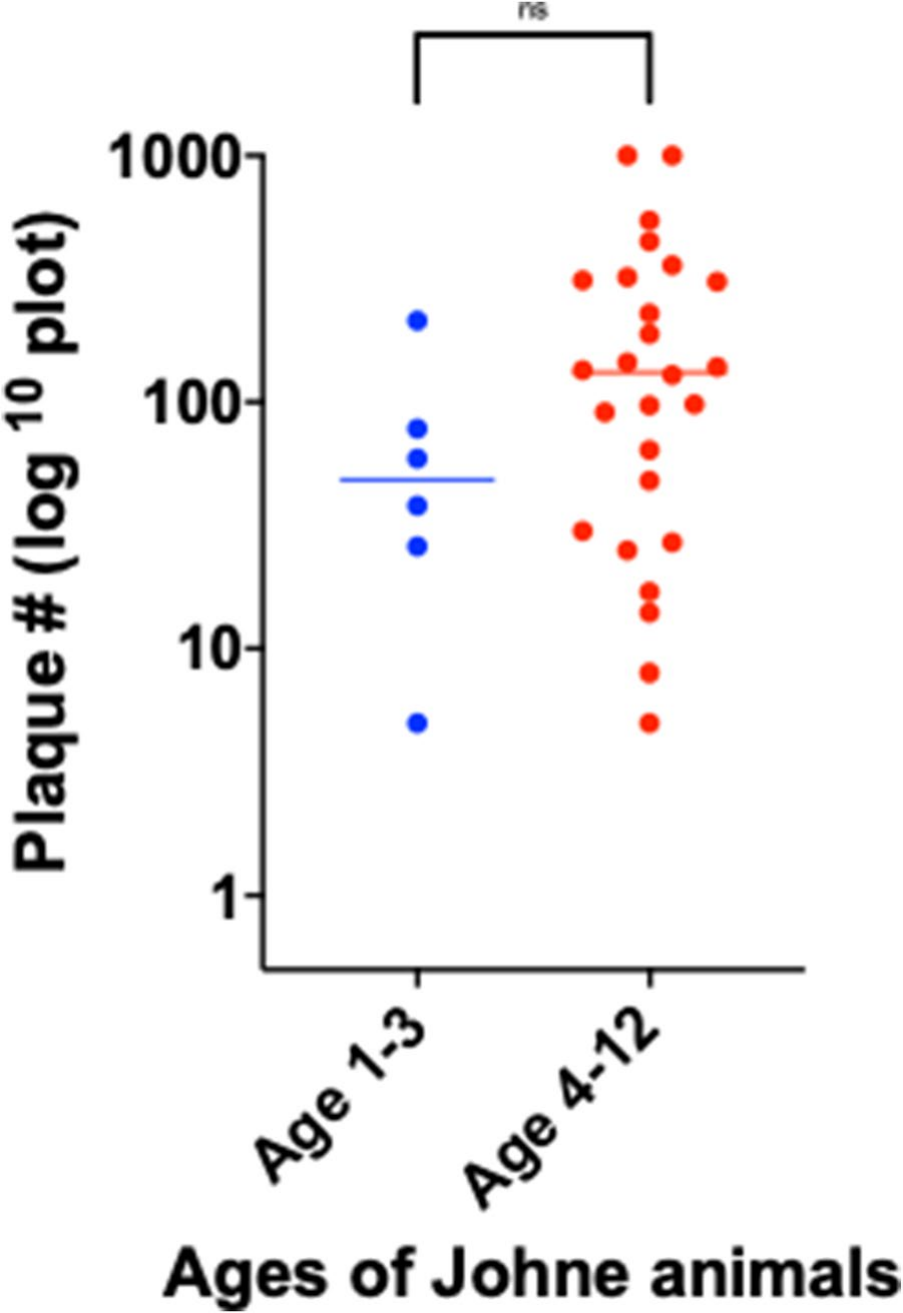
When Johne animals are stratified by ages 1–3 (19%; 6/32) and 4–12 (81%; 26/32) years, the trend for the younger animals to have fewer plaques (70 ± 31) than the older animals (223 ± 53: Mean ± SEM) does not achieve statistical significance (p = 0.19; unpaired two tailed t-test)

The ages of the two groups studied were not significantly different: Johne animals (6.4 ± 3.1 years), Control animals (4.6 ± 3.5 years: Mean ± SD). However, when stratified into two age groups of 1–3 years and 4–13 years there was a higher proportion of younger animals in the Control group; Control age 1–3 years = 53% (9/17), Johne age 1–3 years = 19% (6/32) (Table 5). The younger Control animals have significantly fewer plaques than their older Controls (Fig. 5: p = 0.04). In contrast, amongst the Johne animals the trend to have fewer plaques in the younger animals does not achieve statistical significance (Fig. 6).

### Venn analysis comparing phage/qPCR with three conventional tests

**Fig. 7:**
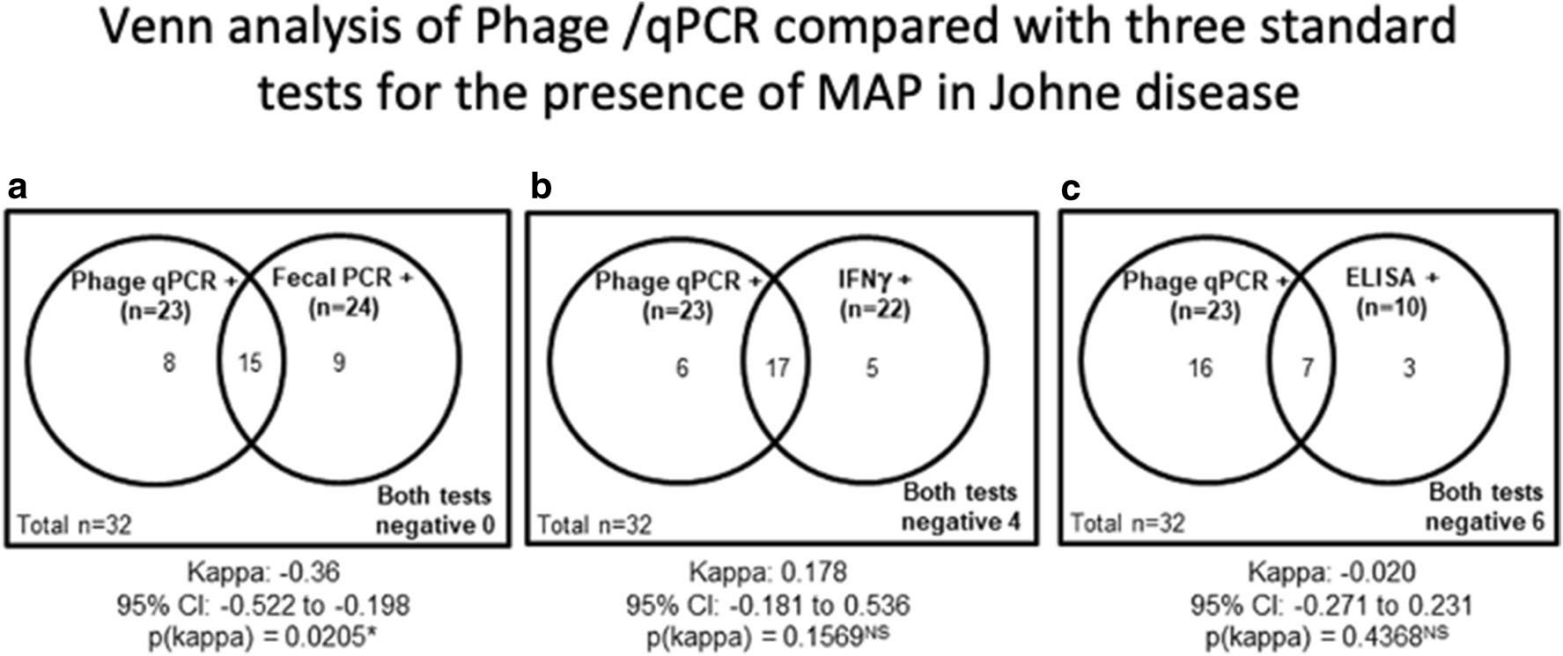
A Venn analysis of the Johne animals, comparing phage/qPCR with the three conventional methods. In combination with fecal PCR, 100% of the 32 Johne animals tested MAP positive, with 15 cows positive in both tests (p(kappa)= 0.02) and no cows testing negative by both tests (**a**). When combined with plasma IFN-γ, there were four cows negative by both tests (p(kappa)  = NS: **b**). When combined with ELISA there were six cows negative by both tests (p(kappa)  = NS: **c**)

A Venn analysis was performed of our phage/qPCR results compared with the three conventional methods (Fecal PCR, IFN-γ and ELISA) in the Johne animals. All (32/32) animals were identified as MAP positive with the phage/qPCR and fecal PCR combined (Fig. 7a). In contrast, 12% (4/32) were MAP negative with combination of phage/qPCR and IFN-γ (Fig. 7b), and 19% (6/32) were MAP negative with combination of phage/qPCR and the ELISA test (Fig. 7c).

## Discussion

We compared the results of the phage/qPCR assay with standard MAP diagnostic modalities (MAP fecal PCR, serum MAP ELISA and plasma MAP IFN-γ). Our data show that when Johne animals were tested the phage/qPCR assay, plasma IFN-γ and fecal PCR are equivalent in terms of percentages of MAP positive samples detected. At the cut-off used for serum ELISA, the detection of prior MAP exposure is less reliable. However, our Venn analysis shows that the combination of phage/qPCR and fecal PCR for MAP identified every Johne animal.

In contrast, the combination of phage/qPCR and either plasma IFN-γ or ELISA (mis)diagnosed 4 and 6 animals as MAP negative, respectively. Thus, the most sensitive combination of tests are the antigen identifying, phage/qPCR combined with fecal PCR. During the course of this study several conventional tests for Johne disease, in Iowa, were elevated in the Control group. However, only a single animal (5c) was moved from the Control herd to the Johne herd. As this is an “Intent-to-Treat” analysis, the data for 5c continues, in this report, to be presented as a Control animal. The other immunological changes were either transient or did not meet the threshold of positivity routinely employed in Iowa infected with MAP at or soon after birth may not manifest any disease until more than a year old and may continue to have productive milk production until age eight. We hypothesize that as the Johne disease becomes more manifest in animals originally in the Control herd, that the normal values of plasma IFN-γ or ELISA will eventually rise above the threshold for a positive test. Accordingly, we posit that this phage/qPCR assay may enable animals to be identified earlier in the course of infection, which could result in more effective preventative interventions.

Our observations differ from prior studies, where Control animals were all MAP negative [23, 26]. In this study, 35% of the Control animals were phage/qPCR positive. This is an indication that despite meticulous efforts of USDA farm staff to separate the control animals from those infected and provide an uncontaminated environment, some Control animals have been exposed to viable MAP and become colonized and/or infected.

The significantly fewer number of plaques in younger Control animals, is an intriguing observation. It may be ascribed to one of several factors. Possibly indicating a more robust immune response in the younger animals. Alternatively, it may be an indication that the tardy rate of replication of MAP might not manifest until animals have lived long enough for the colonizing/infecting MAP to replicate sufficiently to be more easily detected.

Not all mycobacteriophages identified as “D29” act identically in this assay. One strain could not be satisfactorily killed by the viricide (FAS) we use. Considerable time effort and expense was required to determine that it was the D29 and not the FAS viricide we used that was responsible. To date we have not identified why one strain could not be completely killed. We conclude that any investigator attempting to replicate our data should ensure that any strain of D29 used can appropriately be killed by the viricide they employ.

Our observation that apparently healthy control animals have circulating viable MAP should not be surprising. A competent immune response may be able to successfully cope with pathogen exposure. In humans, the presence of viable mycobacteria does not necessarily indicate active disease. One quarter of humankind (~ 1.9 billion people) have latent tuberculosis [28]. However, less than 6% (10 million persons) have active tuberculosis [29]. The DNA of *M. leprae* is found in 5% (calculated to be ~ 70 million) of nasal swabs in the subcontinent of India [30]. However their number of clinical cases of leprosy is ~ 400,000 [31]. Likewise, the failure to identify MAP by qPCR in some animals with clinical Johne is evocative of the inability to identify *M. leprae* in the majority of cases of tuberculoid leprosy [32, 33].

Corroborative evidence that immune competence is critical in successfully combating mycobacterial exposure is seen with the deleterious effect of genetic defects. A Nramp1 genetic defect is associated with susceptibility to Johne disease in cattle [34, 35], sheep [36], as well as leprosy [37] and tuberculosis in humans [38, 39]. In cattle, the presence of a NOD-2 defect results in a > 3-fold increase in the percentage of cattle that have Johne disease, compared to wild type genome cattle in the same herd [40], (see [41] for review of genetic defects influencing Johne disease in cattle). Likewise, a NOD-2 defect is also associated with an increased susceptibility to lepromatous leprosy [42]. Thus, genetic defects may, in part, explain susceptibility to Johne disease, tuberculosis, leprosy and inflammatory bowel disease and account for the presence of undiagnosed (non-MAP) mycobacteria.

## Conclusions

Contrary to our initial hypothesis, using our phage/qPCR assay, we find viable MAP in a proportion of both Johne (72%) as well as Control (35%) animals. In the Johne herd, our data show that phage/qPCR assay, plasma IFN-γ and fecal PCR all detect a similar percentage of positive animals, albeit not always the same animals. In order to detect all animals in the Johne disease group, the most sensitive combination is phage/qPCR with fecal PCR. Phage/qPCR is exquisitely sensitive in detecting the presence of viable MAP in circulating PBMCs. Viable MAP was detected in 35% of the control animals with phage/qPCR, therefore this test identified additional colonized animals or animals with early or sub-clinical infections compared to the standardized, non-antigen-detecting, serum and plasma IFN-γ MAP diagnostics. These data indicate that the presence of viable MAP in blood does not necessarily signify that an animal must of necessity be demonstrably ill or be MAP positive by standard diagnostic methods. This phage/qPCR assay rapidly identifies MAP that must be viable. It has the potential to become a powerful tool in studying the prevalence and penetrance of MAP in human and veterinary diseases.

## Materials and methods

This study was approved by the Research & Development Committee of the James J. Peters VAMC Bronx NY (0720-07-035, MIRB 01002) and Material Transfer Agreement 16432 from USDA Ames Iowa.

### Animals studied

Blood samples were obtained from a herd of naturally MAP infected cattle and their (nominally) non-infected controls [22]. These animals are maintained as part of a small well-characterized group of naturally infected dairy cows and noninfected controls on site at the National Animal Disease Center, Ames, Iowa. Infected and noninfected cows are housed in separate free-stall areas of the dairy barn and all handling and milking is performed separately, to maintain the noninfected controls in a disease-free state as much as possible. The Johne animals were not significantly older (6.4 ± 3.1 years) than the Control animals (4.6 ± 3.5 years: Mean ± SD). All animal related procedures were approved by the IACUC (National Animal Disease Center, Ames, Iowa).

### Routine studies performed

The animals are monitored and regularly and repetitively tested using standard diagnostic methods [22, 43, 44]. In brief these Holstein dairy cattle range in age from 1 to 13 years. Infection is monitored bacteriologically for the fecal shedding of MAP using established methods [17]. Serologic tests used include serum ELISA assayed for MAP antibodies (Herdchek, IDEXX, Westbrook, ME) and plasma bovine IFN-γ (Bovigam test kit: Prionics, La Vista, NE). Animals categorized as infected had ELISA antibody titers averaging 0.48 S/P ratio and antigen-specific IFN-γ results presented as Abs_450nm_ MPS-Abs_450nm_ NS averaged 0.56 ± 0.13. Absorbances greater than 0.1 (Abs_450nm_ MPS-Abs_450nm_ NS > 0.10) were deemed to be positive responses.

### Receipt of samples

Blood samples were drawn into vacutainer tubes containing both Sodium-citrate as the anti-coagulant and Ficoll (Becton-Dickinson: Vacutainer® CPT™ Cell Preparation Tube with Sodium Citrate (8 ml), Cat #362761). Samples were drawn on a Monday, and couriered overnight (FedEx), arriving the following day, usually by 10:30 AM. The phage assay is set up the day that samples arrive. PBMC fraction was harvested by centrifuging vacutainer tubes at 1650×*g* for 30 min and carefully transferring the buffy coat fraction into a new tube containing 1× PBS Buffer.

Because of the intricacies involved in the phage component of the assay, in the initial stages of establishing the assay, we decided to only process blood from four animals at one time for the plaque component of the assay. From 3-17-2019 until 1-16-2020 we received 24 shipments of samples from 32 Johne animals and 17 Control animals, for our phage / qPCR assay. Each animal was studied at least once, and if interesting results, such as a plethora of plaques but negative IS900 qPCR, were studied repetitively. For comparison with our data, the USDA subsequently provided, ELISA, IFN-γ and Fecal PCR results for these animals

### Establishment of MAP phage assay

The optimized MAP phage assay was carried out as described [20, 27]. Critical to the success of the assay is the ability to kill any mycobacteriophages that are not internalized into the mycobacterium being identified. Any unincorporated mycobacteriophages will result in the presence of plaques due to D29 phage plaques due to failure to kill unincorporated D29 and not caused by viable mycobacteria. This is achieved by means of a virucide treatment with Ferrous ammonium sulfate (FAS). Incubation was carried out in Capitol Vial Veterinary Specimen Collection Tubes (ThermoFisher® Scientific: Cat # CNLL2000) the viricidal solution was added, inverted to ensure coating the entire inner surface, incubated at room temp for 5 min, vortexed (3200 RPM: Vortex Mixer OHAUS Cat # VXMNFSEU) for ~20 s and then further incubated for 5 min at room temperature prior to proceeding to plating as described [20]. In every assay, three negative control plates are set up. Conventionally, up to 10 plaques in the control plates are considered as indicating an adequate “negative” control (Fig. 1a).

The mycobacteriophage used in these experiments is D29. Originally isolated from soil in 1954, D29 was initially shown to infect and destroy the *Mycobacterium tuberculosis* bacillus [45]. Subsequently D29 was shown to be active against a variety of mycobacteria including MAP [27]. Two strains were evaluated in these experiments; both labelled “D29”. One was obtained from the University of Pittsburgh USA (a gracious gift of Prof. G. Hatfull). Because of an inability to obtain satisfactory negative controls, a second D29 strain, provided by Prof. Irene Grant of Queens University Belfast (QUB), was evaluated (Fig 1a). The QUB D29 phage had originally been a gift from Dr. Ruth McNerney at the London School of Hygiene and Tropical Medicine UK in 2006.

Plaque count comparison was performed using two amounts of PBMCs isolated from 8 ml blood (10% and 25%) and the plaque counts were compared. Previous studies used 25% [27]. In our studies 25% (4/16) of Controls and 35% (11/32) Johne either had plaque numbers that were “Too Numerous To Count” (TNTC) or the plates were “Cleared” when 25% of the sample was tested; rendering them unquantifiable. Plaques were quantified by visually identifying them on a Stuart Colony Counter (Tequipment Inc.). Accordingly, we present data from the plates inoculated with 10% of the isolated PBMC’s. Where there were no plaques in the 10% PBMC plates, the data were presented as 2/5 of the 25% of PBMC plates. These are identified in the relevant Table as “cal”; for “calculated”. When “TNTC occurred in 10% plates, we estimated the numbers as 1000; identified as “est” in the relevant Table.

### Isolation of DNA

In order to obtain mycobacterial DNA, 20 plaques from each phage assay agar plate were excised and pooled, before DNA was extracted using QIAquick® Gel Extraction Kits (Qiagen) as described by the manufacturer. The extraction was in a final volume of 50 µl. Each qPCR reaction requires 5µl of reconstituted extracted DNA. Eventually in each qPCR assay, samples from a given animal was assayed in quadruplicate. A single positive qPCR result was sufficient to have us declare that animal to be viable MAP positive.

### Strategies to minimize qPCR contamination

In order to minimize the possibility of qPCR DNA contamination, the different components of the assay were performed in physically separated locations. The plaque assay was performed on one floor (room 4F-69). Plaques were harvested and their DNA extracted at separate benches of that one laboratory. Reconstituted DNA was taken to a different floor of the Research block (5F-31). In that location, no mycobacterial assays or culture work is performed. Using separate, UV sterilized, instrument and personal protective clothing the assay was set up using the qPCR plates that were centrifuged in equipment not exposed to any other mycobacterial cultures or nucleotides. The plates are sealed in this location and transferred to a third location (room 3F-33) for qPCR. The plates are sealed prior to being transported for amplification and the seal is never broken once amplification has occurred. The amplified sealed plates are then discarded according to GLP standards, compliant with all relevant regulations for disposal of biological materials.

### qPCR procedures

qPCR was performed using a QuantStudio™ 12K Flex Real-Time PCR System (Applied Biosystems ) with their Multi-Well Plates and Array Card. Primers used were IS900-specific IS900 QF/QR primers (QF:CCG GTA AGG CCG ACC ATT A/ QR:ACC CGC TGC GAG AGC A) and IS900 QP probe (6FAM-CATGGTTATTAACGACGACGCGCAGC-MGBNFQ) [46]. The qPCR assay had subsequently been optimized at Queen’s University Belfast to achieve a sensitivity of 10 genome copies (A Foddai: Personal communication.) Comparisons were made between two quenchers: recommended TAMRA [27], and MGB (recommended by Applied Biosystems) (Fig. 2). Because of the increased fluorescence with MGB, this was the quencher used for our experiments.

### Statistical analysis of results

Statistical analyses were performed using Prism 8 (https://www.graphpad.com). A Venn comparison was performed on 2×2 contingency tables. The Kappa and p value agreement between results of the phage / qPCR assay and the three other standard tests was calculated using EpiTools (https://epitools.ausvet.com.au/comparetwotests). Significance for all statistical analyses was accepted at p ≤ 0.05.

## Data Availability

All presented in this manuscript.

## Abbreviations

MAP: Mycobacterium avium subspecies paratuberculosis
PBMC’s: Peripheral blood mononuclear cells
IFN-γ: Interferon-γ
USDA: United States Department of Agriculture
qPCR: Quantitative polymerase chain reaction amplification
FAS: Ferrous ammonium sulfate
PFU: Plaque Forming Units
